# Association of preoperative Circulating biomarkers with echocardiographic measures of right ventricular strain five years after tetralogy of fallot repair

**DOI:** 10.1007/s10554-025-03533-4

**Published:** 2025-10-27

**Authors:** Jose A. Galan, Jennifer A. Faerber, Andrea L. Jones, Ryusuke Numata, Angeli Thomas, Anh Duc Mai, Yan Wang, Adam S. Himebauch, Monique M. Gardner, Laura Mercer-Rosa

**Affiliations:** 1 Children’s Hospital of Philadelphia, Philadelphia, PA USA; 2 Levine Children’s Hospital, Charlotte, NC USA; 3 University of Pennsylvania, Perelman School of Medicine, Philadelphia, PA USA; 4 Division of Cardiology, Children’s Hospital of Philadelphia, 3400 Civic Center Boulevard, Suite 8NW35, Philadelphia, PA 19104 USA

**Keywords:** Tetralogy of fallot, Biomarker, Echocardiography, Right ventricle, Strain, Congenital heart disease

## Abstract

**Purpose:**

We analyzed right ventricular (RV) function using echocardiography after tetralogy of Fallot (TOF) repair to investigate the association between preoperative circulating biomarkers and RV strain five years after repair. We hypothesized that biomarkers of myocardial fibrosis and stretch would be correlated with worse mid-term RV function measured by strain.

**Methods:**

We included children repaired at our institution from 2016 to 2019. Biomarkers measured preoperatively included galectin-3, Procollagen-type-I carboxy-terminal-propeptide (PICP), Procollagen-type-III-amino-terminal-propeptide (PIIINP), matrix-metalloproteinase-1 (MMP1), matrix-metalloproteinase-9 (MMP9), soluble suppression of tumorigenicity-2 (sST2), and N-terminal-pro-B-type-natriuretic-peptide (NT pro-BNP). Function was retrospectively assessed with RV longitudinal peak systolic strain (RVLS), free-wall peak systolic strain (FWS), peak systolic strain-rate (SR) and free-wall systolic strain-rate (FWSR) on available echocardiograms from one to five years after repair. Multivariable linear regression models with Generalized Estimating Equations tested whether preoperative biomarkers were associated with RV strain.

**Results:**

In total, 56 patients were followed with 198 reviewed echocardiograms, with a median of 4 echocardiograms per patient (range 1–5). Strain parameters remained stable until year 2 followed by a significant decrease in all parameters by year 3 through year 5. Higher preoperative levels of MMP1, PICP and PIIINP were associated with worse RVLS. Higher NT-proBNP was associated with better FWSR.

**Conclusion:**

RV function is dynamic in the first 5 years after TOF repair but remains stable in the mid-term. Preoperative biomarkers of extracellular matrix turnover could help explain postoperative RV function in patients with TOF.

**Supplementary Information:**

The online version contains supplementary material available at 10.1007/s10554-025-03533-4.

## Introduction

The unrepaired right ventricle (RV) in tetralogy of Fallot (TOF) is subject to significant stressors such as cyanosis and pressure overload which can lead to myocardial fibrosis. Research on histopathological specimens has identified preoperative fibrosis as a factor influencing postoperative RV function assessed by tissue Doppler imaging [1]. We have previously demonstrated the association of biomarkers of myocardial fibrosis with RV strain early in repaired TOF (rTOF) [2]. Additionally, several studies have demonstrated that circulating biomarkers might be valuable screening tools for identifying and predicting patients at risk for adverse outcomes after heart surgery [3–6].

Despite excellent operative survival rates, TOF repair often results in pulmonary regurgitation, and less commonly in right ventricular outflow tract obstruction, both of which, if left untreated, lead to hemodyamic overload of the RV with subsequent RV dysfunction and eventual failure, resulting in adverse long-term outcomes including greater mortality risk as patients transition from adolescence into adulthood [7, 8]. The time at which the RV remodeling (changes in RV shape, size, and function) transitions from “adaptive” to “maladaptive” with a decline in RV function remains unknown. For the purposes of this paper, we will refer to remodeling as maladaptive unless otherwise specified.

Echocardiography plays an essential role in the follow-up of these patients to evaluate residual lesions and monitor for RV remodeling. Speckle-tracking echocardiography is a sensitive method to assess RV systolic function. RV longitudinal and free wall strain measurements are relatively preload- and afterload-independent and represent an important aspect of echocardiographic TOF evaluation [9] as it may detect subclinical changes in function, predicting adverse outcomes in patients with heart failure [10]. In a different patient cohort we have previously demonstrated that RV strain worsens early after TOF repair and improves within the following two years [11] however, research on remodeling over the first five years after repair (mid-term) remains limited and could help understand post-TOF repair events occuring during childhood [12].

To our knowledge, no studies have investigated whether preoperative circulating biomarkers are associated with mid-term RV remodeling. In this study, we sought to analyze RV remodeling after TOF repair and evaluate the association between preoperative biomarkers and mid-term RV function measured by strain in TOF. We hypothesized that biomarkers of myocardial fibrosis and stretch would be associated with worse mid-term RV function measured by strain.

## Methods and materials

### Study design and population

We conducted a retrospective analysis of data obtained as part of a prospective single center cohort study of patients with TOF who underwent complete repair at Children’s Hospital of Philadelphia (CHOP) between September 2016 and September 2019. Children who underwent palliative procedures prior to complete repair (e.g.: placement of an aorto-pulmonary shunt or stent in the ductus arteriosus) were also included. The study cohort has been previously described [2, 13, 14]. We included patients with preoperative biomarker measurements. Subjects without any postoperative echocardiograms were not included in the mixed-effects models because they provide no information on outcome trajectories therefore were excluded. Complete TOF repair was defined as closure of the ventricular septal defect (VSD) with relief of RV outflow tract obstruction when necessary. We excluded patients who completed the TOF repair after two years of age.

The study protocol was approved by the Institutional Review Board of Protection of Human Subjects at Children’s Hospital of Philadelphia (CHOP) IRB: 23–021676, and the parents of the subjects provided informed consent to participate in the study.

### Clinical data

Clinical variables including demographic and perioperative characteristics, cardiac history, and operative variables were abstracted from the medical records and available for analysis.

### Biomarkers

Seven biomarkers were measured based on published data in children and adults with congenital heart defects: Galectin-3, procollagen type-I carboxy-terminal propeptide (PICP), procollagen type-III amino terminal propeptide (PIIINP), matrix metalloproteinase 1 (MMP1), matrix metalloproteinase 9 (MMP9), soluble suppression of tumorigenicity 2 (sST2), and N-terminal pro B-type natriuretic peptide (NT pro-BNP) [3–5, 15]. Blood samples were obtained at the time of TOF repair before cannulation for cardiopulmonary bypass. sST2 was only collected in the first 46 patients. Fifty-six (74%) patients had PICP levels greater than the maximum level of detection (6400 ng/ml), thus a binary variable (high/low PICP) was created. High PICP was defined as >6400 ng/ml and low PICP was defined as < 6400 ng/ml.

### Biomarker assays

Serum and EDTA plasma samples were processed and frozen at − 80 ◦C for further analysis by the biomarker core laboratories from the Translational Core at CHOP for NT-proBNP, Galectin-3, sST-2, MMP-1, and MMP-9. Biomarkers were assayed using commercially available reagents by personnel who were blinded to clinical status.

Galectin-3 was measured in triplicate on the ELLA system. The average coefficient of variation (CV) was 1.68% (range 0.18–9.59%, *n* = 133). Inter-plate CV was 1.24% for QC1 (mean = 26.3 pg/ml, range = 22.3–37.2 pg/ml) and 2.10% for QC2 (mean = 1322 pg/ml, range = 1195–1992 pg/ml).

MMP-1 and MMP-9 were measured using a 2-plex kit also from MSD (Cat#: K15034C-2). Samples were tested in duplicate and the mean CV was 3.48% for MMP1 and 3.47% for MMP9 (range MMP1 = 0.17–9.80%, range MMP9 = 0–9.84%; *n* = 133). Inter-plate CV for QC1 was 8.43% for MMP-1 (mean = 89.3 pg/ml, range = 75.0–125 pg/ml) and 9.43% for MMP-9 (mean = 508 pg/ml, range = 375–625 pg/ml); for QC2 the inter-plate CV was 6.19% for MMP-1 (mean = 918 pg/ml, range = 750–1250 pg/ml) and 1.42% for MMP-9 (mean = 4565 pg/ml, range = 3750–6250 pg/ml); the inter-plate CV for QC3 was 6.57% for MMP-1 (mean = 9232 pg/ml, range = 7500–12,500 pg/ml) and 6.04% for MMP-9 (mean = 41,326 pg/ml, range = 37,500–62,500 pg/ml). Soluble ST2 was measured using the ELLA Automated Immunoassay System (Protein Simple; San Jose, California). Samples were tested in triplicate, and the mean CV was 1.91% (range = 0.21–7.56%, *n* = 133). Inter-plate CV was 0.60% for QC1 (mean = 365 pg/ml, range = 254–423 pg/ml) and 1.46% for QC2 (mean = 14,285 pg/ml, range = 12,679–21,131 pg/ml).

NT-proBNP was measured using kits (Cat#: K151JKC-2) from the Meso Scale Discovery (MSD; Rockville, Maryland). Samples were tested in duplicate and the mean CV was 5.43% (range = 0.01–9.90%, *n* = 138). Inter-plate CV was 7.21% for QC1 (mean = 11.4 pg/ml, range = 7.50–12.5 pg/ml), 6.52% for QC2 (mean = 89.3 pg/ml, range = 75.0–125 pg/ml), and 4.93% for QC3 (mean = 997.5 pg/ml, range = 750.0–1250 pg/ml).

### Echocardiograms

**Fig. 1:**
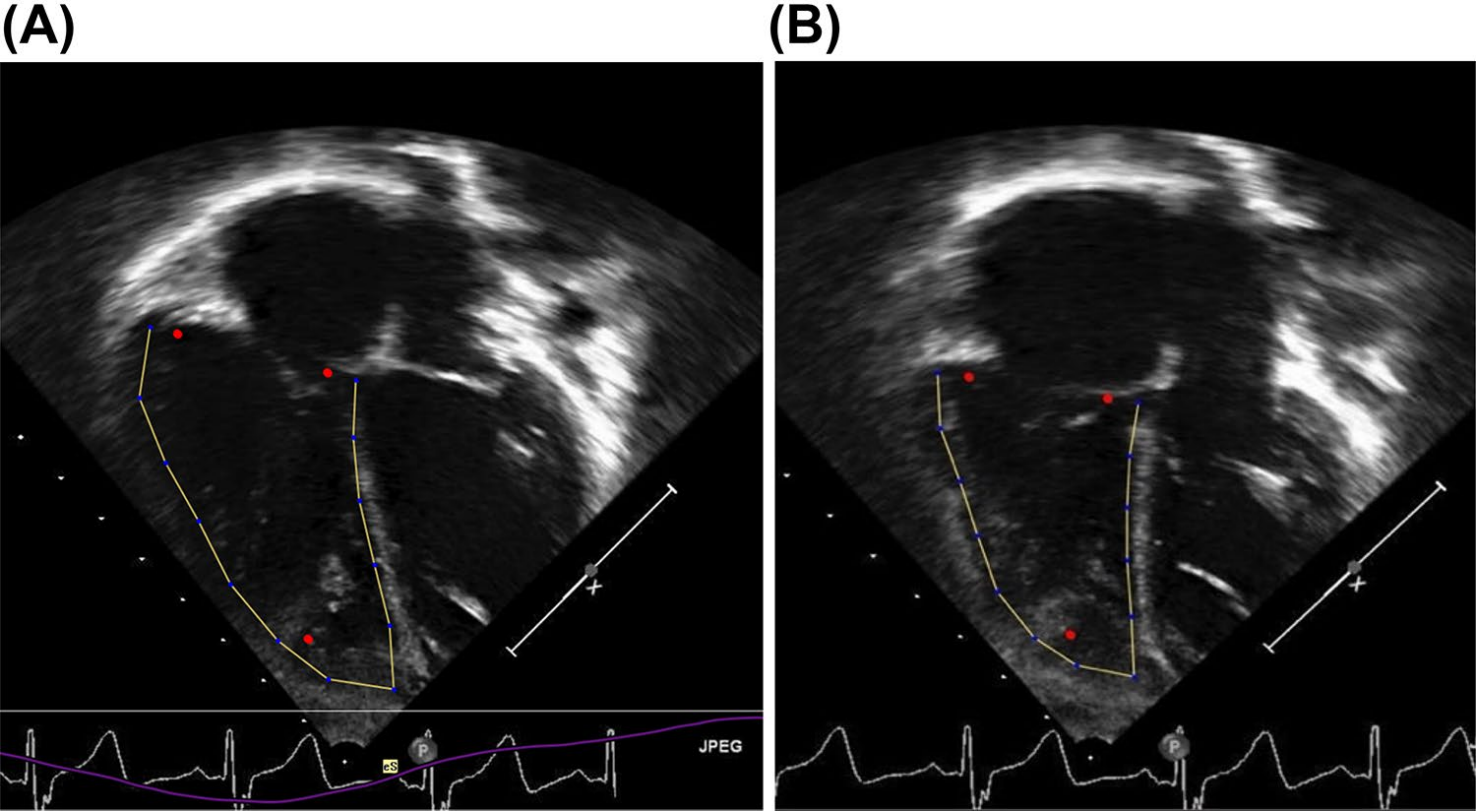
Echocardiographic measurements. Echocardiographic four-chamber view. The RV endocardium was traced at end-diastole (**A**) and end-systole (**B**) (Yellow boundaries)

RV strain was measured from echocardiograms obtained during the typical yearly routine outpatient visit using uncompressed data. We examined up to five years of follow-up data per patient. Images closest to the year mark were selected and considered suitable if the entire RV (free wall, apex and septum) were within the frame for at least one complete cardiac cycle on apical four chamber images, and if the frame rate was between 50 and 95 frames/second (Fig. 1**)**. Covariates “RV dilation” and “pulmonary stenosis” were defined by an end-diastolic area (EDA) Z-score > 2, and as a peak velocity across the pulmonary valve > 2.5 m/s respectively. Echocardiograms with measurable strain images were available for 74% of patients in year 1, 60% in year 2, 44% in year 3, 48% in year 4, and 37% in year 5.

RV longitudinal peak systolic strain (RVLS), free wall longitudinal peak systolic strain (FWS), RV peak systolic strain rate (SR) and free wall peak systolic strain rate (FWSR) were assessed tracing the endomyocardium with speckle tracking analysis using TomTec software (Cardiac Performance Analysis, TomTec, Germany) by a single observer (JAG). Tracings were generated automatically by the software and subsequently adjusted manually for optimal tracking. Prior to initiating data collection, intra-reader reliability was assessed using intraclass correlation coefficients (ICC) derived from two measurements across 10 distinct samples. Subsequently, inter-reader reproducibility was evaluated using ICC based on 10 randomly selected samples measured independently by a second observer (RN). For ease of interpretation, worsening RV function was defined as a decrease in the absolute value of strain (typically a negative value), and improvement in function as an increase in the absolute value of strain.

### Statistical analysis

Descriptive statistics were calculated as frequency counts and percentages for categorical variables and mean ± standard deviation (SD) or median and interquartile range (IQR) for continuous variables. The preoperative biomarkers constituted the exposure of interest, and outcomes were strain measures over time. Covariates and potential confounders included patient and surgical factors, including type of TOF surgical repair and presence of a genetic syndrome.

Associations between exposures and outcomes were analyzed with univariable and multivariable linear regression models with Generalized Estimating Equations (GEE). Multivariable linear mixed-effects (LME) models were run as sensitivity analyses. The primary models were used to test whether preoperative biomarkers were associated with the postoperative strain measures using up to 5 years of follow-up echocardiographic measurements per patient. Each of the four strain outcomes (RVLS, FWS, SR, and FWSR) was analyzed in regression models with each biomarker, time of measurement, and preoperative strain as covariates. First, preoperative strain and time were used as covariates to explore the relationship of biomarkers with postoperative function, unadjusted for patient covariates (GEE Model 1). Then we added clinically important covariates which could be potential confounders in the adjusted model (GEE Model 2) including included type of TOF repair (transannular patch, RV to pulmonary artery conduit, non-transannular patch, VSD closure only) and diagnosis of a genetic syndrome.

To correctly specify the correlation structure for the GEE and LME models, we tested with four correlation structure functions, including: *autoregressive*,* exchangeable*,* independence and unstructured*. We chose the correlation structure with the lowest Quasi-likelihood under Independence Model Criterion (QIC value). We calculated the false discovery rate using the Benjamini-Hochberg procedure to account for all biomarker-outcome models, though we ran these models four different ways: (1) fixed effects unadjusted for patient covariates, (2) fixed effects adjusted for patient covariates, (3) GEE regression model and (4) LME regression model to check the conclusions reached by the primary models.

For the biomarkers with an identified linear relationship with the strain outcome, we also checked for nonlinear associations by fitting restricted cubic splines with three knots at percentiles of the biomarker distribution. We checked to see if the nonlinear model fit the data better than the linear model using goodness of fit statistics (e.g. QIC and QICu). We found significant nonlinear associations between MMP1 and RVLS, but the goodness of fit statistics were lower for the linear model than the model with restricted cubic spline terms, indicating that the linear model was preferrable for MMP1. No other significant nonlinear associations between biomarkers and strain were identified.

### Additional analyses

We used the same statistical methodology to assess changes in strain over time using time as the exposure instead of preoperative biomarkers. We ran linear regression models with GEE to test if there were a categorical effect of each post-repair year compared to the first-year data.

In sensitivity analyses, we assessed whether time-dependent variables—right ventricular (RV) dilation and residual pulmonary stenosis—might impact the relationship between biomarkers and strain. To minimize bias from time-dependent variables, we applied the framework by Burcu & Oehrlein [16] and adjusted separately for the presence of RV dilation and pulmonary stenosis in the multivariable GEE models (Online supplement). Additionally, RV EDA Z-score (treated as a continuous variable) was not significantly associated with strain and was not included in final adjusted models. Full sensitivity model results are provided in the Online Supplement.

All analyses were performed using SAS Software version 9.4 (SAS Institute, Cary, NC). We considered a p-value of < 0.05 to be statistically significant. Given completeness of patient characteristics, there was no missing data imputation performed in this study.

## Results

### Patient characteristics

**Fig. 2:**
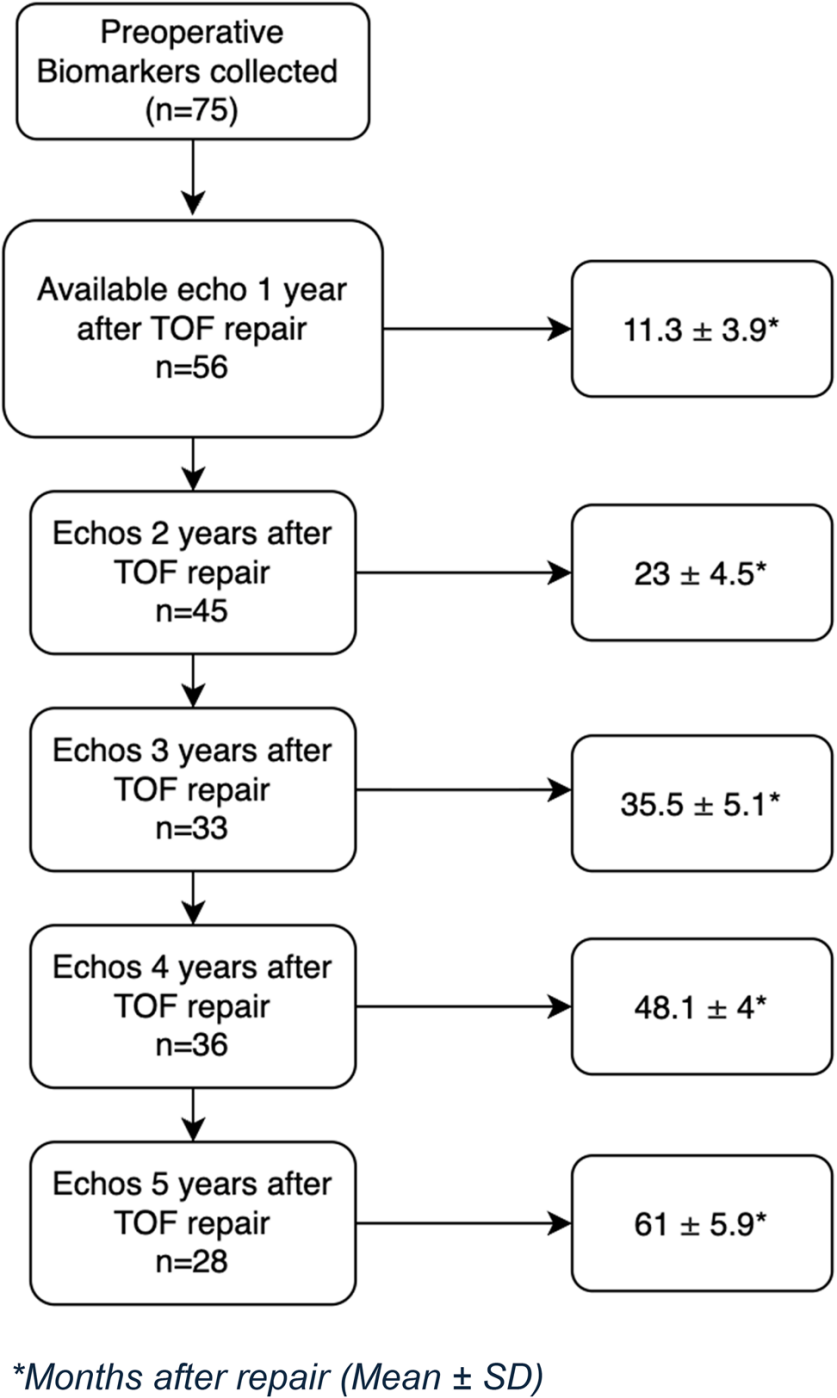
Study population. *Months after repair (Mean ± SD)

Fifty-six patients with preoperative biomarkers and echocardiographic measurements were included in the study, most were male (61%) and White (73%). The most common pulmonary valve anatomy was pulmonary stenosis (89.3%) and 58.6% of the patients received a transannular patch surgical repair. Two patients died during follow up and 17 were lost in follow up with no echocardiorgams after 1 year. (Table 1; Fig. 2)

**Table 1:** Patient and operative characteristics of the study cohort

*N* = 56	Median [IQR] or *n* (%)
Age at repair (months)	3.7 [2.1–5.1]
Male	37 (66%)
White	45 (80%)
Hispanic or Latino	7 (12.5%)
Palliative procedures	16 (28.5%)
Genetic Diagnosis	20 (35.7%)
*Pulmonary valve anatomy*	
Pulmonary stenosis	50 (89.2%)
Pulmonary atresia	5 (8.9%)
Absent pulmonary valve	1 (1.7%)
*Type of repair*	
Transannular patch	33 (58.9%)
Non transannular	5 (8.9%)
RV to PA conduit	8 (14.2%)
Pulmonary Valvotomy	4 (7.1%)
No RVOT augmentation	6 (10.7%)
*Access for VSD closure*	
Transatrial	48 (85.7%)
Ventriculotomy	8 (13.3%)
Total CPB Time (minutes)	72 [39–94.2.2]
Length of stay after TOF repair (Days)	6 [5–9]

RVOT (Right ventricular outflow tract), VSD (Ventricular septal defect), CPB (Cardiopulmonary bypass)

### Strain measurements

**Fig. 3:**
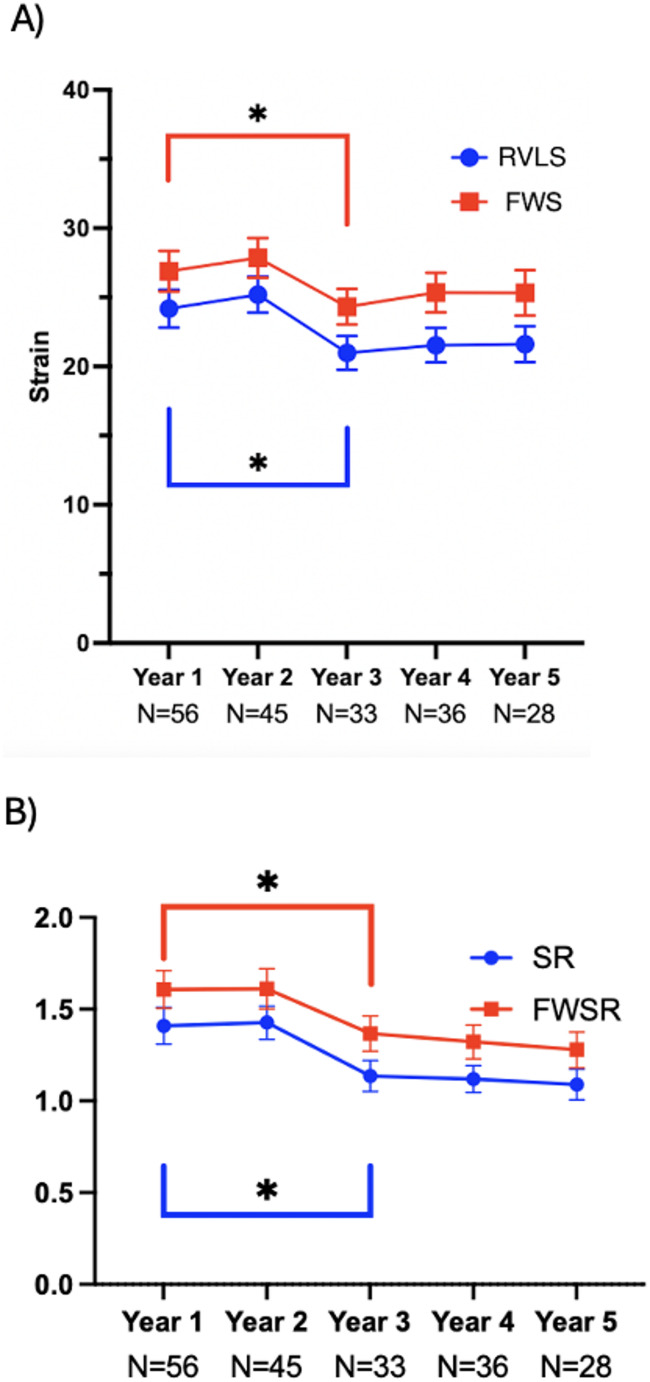
RV strain measures over time. Each dot represents the mean of individual values recorded at each year of follow-up and 95% confidence intervals displayed for each time point. (A)Strain measurements are reported as percentages (%), (B) while strain rate is expressed in s⁻¹. Compared to Year 1, there was a significant worsening in RVLS, FWS, SR, and FWSR by Year 3 (*p < 0.05), but no further deterioration was observed between Years 3, 4, and 5.

In total, 198 echocardiograms were suitable for analysis. Intra- and inter-reader ICCs were 0.92 (95% CI: 0.84,0.98) and 0.96 (95% CI: 0.89,0.98), and the absolute RVLS mean difference between observers was 1.5, confirming reproducibility. RV strain measurements peaked in year 2 following TOF repair. Compared to year 1 postoperative values of RVLS, FWS, SR and FWSR remained stable in year 2. This was followed by a significant decrease in all RV strain parameters in year 3, with no further worsening, through year 5. (Fig. 3; Table 2).

**Table 2:** Mixed effect model results comparing changes of RV function over time

	Year 1 (*n* = 56)	Year 2 (*n* = 45)					Year 3 (*n* = 33)				Year 4 (*n* = 36)			Year 5 (*n* = 28)			
	Reference (95% CI)	Mean ± SD		Beta (95% CI)	P		Mean ± SD	Beta (95% CI)	P		Mean ± SD	Beta (95% CI)	P	Mean ± SD	Beta (95% CI)		P
RVLS	23.8 (22.4, 25.3)	25.1 4.3		1.3 (−0.2, 2.9)	0.1		20.9 3.4	**−2.8** **(−4.4**,**-1.3)**	**0.0002**		21.5 3.6	**−2.3** **(−3.8**,** −0.8)**	**0.0022**	21.6 3.3		**−2.2** **(−3.7**, **−0.72)**	**0.0039**
FWS	26.5 (25.09, 28.08)	27.8 4.7		1.2 (−0.5, 3.02)	0.14		24.3 3.6	**−2.2** **(−3.9**,** −0.5)**	**0.0089**		25.3 4.2	−1.2 (−3.09, 0.5)	0.1	25.3 4.2		−1.2 (−3.1, 0.6)	0.1
SR	1.39 (1.29, 1.49)	1.4 0.30		0.03 (−0.07, 0.13)	0.57		1.1 0.23	**−0.25** **(−0.36**,** −0.15)**	**< 0.0001**		1.13 0.21	**−0.27** **(−0.38**,** −0.16)**	**< 0.0001**	1.08 0.21		**−0.30** **(−0.41**, **−0.20)**	**< 0.0001**
FWSR	1.5 (1.48, 1.69)	1.6 0.36		0.019 (−0.09, 0.13)	0.72		1.36 0.27	**−0.22** **(−0.34**,** −0.10)**	**0.0003**		1.32 0.27	**−0.26** **(−0.39**,** −0.14)**	**< 0.0001**	1.27 0.24		**−0.3** **(−0.43**,** −0.19)**	**< 0.0001**

Bold values represent statistical significance (*p* < 0.05)

Strain measurements are reported as percentages (%), while strain rate is expressed in s⁻¹. Compared to Year 1, there was a significant worsening in RVLS, FWS, SR, and FWSR by Year 3 (*p* < 0.05), but no further deterioration was observed between Years 3, 4, and 5

### Primary outcome models

We identified associations between preoperative biomarkers and postoperative RV function in both unadjusted (for additional patient covariates) (GEE Model 1) and adjusted (GEE Model 2) linear regression models (Table 3). In unadjusted GEE Model 1, elevated levels of MMP1 and PIIINP were associated with worse mean values of postoperative RVLS, and elevated values of MMP1 were associated with worse mean values of postoperative FWS. Conversely, higher levels of NT pro-BNP were associated with average higher values of RVLS, FWS, SR and FWSR. After adjusting for covariates (GEE Model 2), Higher MMP1, PIIINP and PICP levels were significantly associated with worse RVLS, whereas elevated levels of MMP1 were associated with worse FWS. Higher levels of NT-proBNP were associated with better FWSR. There were no significant associations with average strain parameters for Galectin-3, MMP9, and ST-2 in either model. In the additional GEE model adjusting for either RV dilation or pulmonary stenosis (see Online Supplement), MMP1, PIIINP, and elevated PICP remained significantly associated with worse RVLS. And the association between MMP1 and FWS also persisted when adjusting for pulmonary stenosis, In contrast, other previously observed associations with FWS and FWSR were not sustained (the relationship between MMP1 and FWS was no longer significant after adjusting for RV dilation, and NT-proBNP was not associated with FWSR after adjusting for either RV dilation or pulmonary stenosis). Results from sensitivity analyses demonstrated that while both RV dilation and pulmonary stenosis were independently associated with strain outcomes, neither were, for the most part, associated with preoperative biomarker levels, thus not confounding the biomarker-strain relatioship.

All the p-values from linear regression models were larger than the critical values calculated from the false discovery rate, so we report on associations identified bearing in mind that some of these relationships might have been observed by chance.

**Table 3:** Associations of each biomarker with strain outcomes

Association	Model 1	Model 2
RVLS	Unadjusted Estimate (95% CI), *P* value	Adjusted Estimate (95% CI), *P* value
MMP1	**−0.0227 (−0.0441, −0.0014) 0.0366**	**−0.0272 (−0.0466, −0.0078) 0.006 **
MMP9	−0.0111 (−0.0271, 0.0048) 0.1701	−0.0082 (−0.0241, 0.0078) 0.3146
ST-2	−0.014 (−0.1223, 0.0942) 0.7997	0.0162 (−0.0697, 0.1022) 0.7109
Gal 3	0.1299 (−0.1648, 0.4245) 0.3877	0.0674 (−0.2274, 0.3623) 0.6541
Nt-Pro BNP	0.3314 − 0.0113 0.6741 0.0581	0.2064 (−0.0866, 0.4995) 0.1674
PIIINP	**−0.2015 -0.3716 -0.0314 0.0203 **	**−0.1774 (−0.3281, −0.0267) 0.0211 **
PICP	0.0003 − 0.0002 0.0007 0.2177	**−1.1685 (−2.2441, −0.093) 0.0332 **
*FWS*		
MMP1	−0.0173 -0.0351 0.0006 0.0578	**−0.0173 (−0.0328, −0.0018) 0.028**
MMP9	−0.0088 -0.0251 0.0075 0.2879	−0.0033 (−0.0194, 0.0128) 0.6878
ST-2	0.0199 –0.0813 0.1211 0.6999	0.0485 (−0.0404, 0.1373) 0.2848
Gal 3	0.1526 −0.145 0.4503 0.3148	0.0908 (−0.2065, 0.3881) 0.5495
Nt-Pro BNP	**0.3471 0.0317 0.6625 0.031**	0.2026( −0.1308, 0.536) 0.2336
PIIINP	−0.1447 -0.3117 0.0223 0.0895	−0.0408 (−1.5366, 1.454) 0.9573
PICP	0.0002 –0.0003 0.0006 0.4655	**−0.0003(−0.0006,0)0.0567**
*SR*		
MMP1	−0.0011 -0.0031 0.001 0.3187	−0.0011 (−0.0031, 0.0009) 0.2632
MMP9	−0.0005 -0.0016 0.0006 0.3516	−0.0002 (−0.0012, 0.0008) 0.7047
ST-2	−0.0031 -0.0097 0.0035 0.3532	−0.0002 (−0.0057, 0.0053) 0.9473
Gal 3	0.0004 –0.0161 0.0169 0.9601	−0.0038 (−0.0204, 0.0127) 0.651
Nt-Pro BNP	0.0197 − 0.0015 0.041 0.069	0.0148 (−0.0056, 0.0352) 0.1554
PIIINP	**−0.0047 -0.0174 0.008 0.4664**	−0.0044 (−0.0165, 0.0077) 0.4777
PICP	0 –0.0001 0 0.0001 0 0.6459	−0.0448 (−0.1401, 0.0504) 0.3565
*FWSR*		
MMP1	−0.0003 -0.0024 0.0018 0.7673	−0.0003 (−0.0024, 0.0018) 0.7789
MMP9	−0.0003 -0.0015 0.0008 0.5655	−0.0012, (0.0012) 0.9703
ST-2	−0.0017 -0.0074 0.0039 0.5501	0.0022 (−0.0021, 0.0065) 0.3175
Gal 3	0.0001 –0.0143 0.0146 0.9864	−0.0037 (−0.0176, 0.0101) 0.5994
Nt-Pro BNP	**>0.0293 0.0063 0.0523 0.0124**	**0.0244 (0.0003, 0.0486) 0.0476**
PIIINP	−0.002 -0.0132 0.0091 0.7221	−0.0017 (−0.0129, 0.0094) 0.7582
PICP	0–0.0001 0 0.00010 0.4976	−0.037 (−0.1534, 0.0793) 0.5324

Bold values represent statistical significance (*p* < 0.05)

For each unit increase in biomarker levels, there was a direct effect on strain, expressed as a percentage (%) or strain rate (s⁻¹)

## Discussion

In this single center cohort study with retrospective analysis of clinically indicated echocardiograms performed in rTOF, we investigated changes in RV systolic function using myocardial deformation measured as strain in the initial 5 years after TOF surgical repair and tested associations between preoperative circulating biomarkers with mid-term RV strain. After an initial 2-year improvement in strain, we identified significant worsening in RV strain starting in year 3 after TOF repair. Higher levels of biomarkers MMP1, PICP and PIIINP were associated with worse RVLS at follow-up, and higher preoperative NT-proBNP levels were associated with better FWSR.

Although RV remodeling in TOF is well described from fetal life until surgical repair [12, 17] and vast literature describes RV remodeling late after rTOF, there is a paucity of data on changes occurring in the initial years following TOF repair, when the RV is exposed to volume and/or pressure overload from residual lesions [18, 19]. We report changes in RV strain in the initial 5 years after TOF repair. Although the literature presents conflicting findings regarding the influence of loading conditions on strain [20, 21], strain remains a more sensitive and reliable marker for detecting subtle changes in myocardial function and predicting adverse outcomes compared to traditional parameters [22]. For this reason, we selected strain as the primary method for quantifying RV function instead of using more standard 2D function parameters like fractional area change. Annavajjhala et al. examined RVLS changes in the initial 8 years after TOF repair depending on the type of repair [23]. They found mid-term RV function improvement in relation to the immediate postoperative period. Similar findings were observed in a separate cohort of patients studied by our group [11], lending credence to our theory of RV remodeling during the initial years following TOF repair. Building on these findings, We examined changes relative to the one-year mark, as RV recovery is typically achieved by that time. Our analysis suggests that recovery may extend into the second year, followed by a decline and subsequent stabilization in RV function without reaching normal levels [24, 25]. We hypothesize that the initial stabilization in RV function over the 2 years following TOF repair represents a recovery phenomenon from surgery itself (ventriculotomy, VSD patch, etc.) and exposure to cardiopulmonary bypass and possibly a compensatory contractile mechanism from volume overload to the right ventricle in the presence of pulmonary insufficiency. It is important to note that this trend in strain is not sustained beyond the second year after repair, which may mark the onset of adverse RV remodeling following surgery, as the RV is subject to residual volume and/or pressure load. Given that the RV appears to adapt within the first years post-repair possibly due to lower afterload and resistance in the pulmonary circulation, we believe that the study of outcomes after TOF repair could benefit from echocardiographic strain assessment.

Associations with RV function were observed for MMP1, PICP, PIIINP, and NT-proBNP. MMP1 is an enzyme responsible for degrading the extracellular matrix (ECM), including collagen [26]. It has been directly associated with left ventricular end-diastolic volumes and inversely associated with ejection fraction in heart failure patients particularly in pressure-loaded ventricles [27]. Other MMPs, such as MMP2, have been associated with worse diastolic function in adolescents and young adults with repaired TOF [28]. We previously found that MMP1 levels were directly associated with RV mass and RV mass-to-volume ratio on cardiac magnetic resonance imaging in older children and adolescents with rTOF [29]. In the present study we hypothesize that the MMP1 association could be explained via a relationship between greater extracellular matrix turnover in a state of hypertrophy in the preoperative pressure-loaded RV. However, we could only demonstrate an association between preoperative levels of MMP1 with worse postoperative RV strain; we could not correlate MMP1 with RV hypertrophy given the lack of cardiac magnetic resonance imaging studies prior to TOF repair, and the limitations to quantify RV hypertrophy on pediatric echocardiograms. These associations suggest a potential involvement of MMP1 related mechanisms in RV remodeling in patients after TOF repair. We are not aware of other studies that have investigated preoperative MMP1 levels and their relationship with postoperative RV function in TOF patients.

PICP and PIIINP are released in the circulation upon degradation of fibrillar collagen type I and III [30] and have been linked with severity of myocardial fibrosis and poor outcomes in patients with systemic hypertension and heart failure [31, 32]. Histopathologic myocardial fibrosis [33], elevated collagen turnover prior to surgical TOF repair and high circulating levels of PICP and PIIINP in TOF patients have been associated with greater extent of focal fibrosis, RV dilation, worse exercise performance, LV and RV systolic function (measured by cardiac MRI) and with lower arterial oxygen saturation prior to surgical TOF repair [4, 34, 35]. Here, we demonstrate that higher levels of preoperative PICP and PIIINP were associated with worse RVLS in the initial years after TOF repair. Although the previous studies do not offer a direct comparison to our study given the age difference among study patients, they support the association of increased circulating pro-collagen biomarkers with RV dysfunction in patients with TOF repair. Reductions in PIIINP have been reported in other CHD patients following ACEI treatment. This underscores the need for future studies to determine whether PIIINP could serve as an endpoint for therapeutic trials targeting postoperative RV remodeling in TOF patients [35, 36].

Higher preoperative NT-proBNP levels were associated with better RV function at follow up. This is in contrast to other studies in rTOF that showed an association of elevated NT-proBNP levels with worse RV function and RV volume overload late after rTOF [37, 38]. A study in children with CHD showed that NT-proBNP levels are generally higher in patients with LV volume overload compared to those with RV pressure overload [39]. Our finding could be explained by the fact that patients in this study had variable degrees of preoperative pressure loading of the RV, since we also included patients with the so-called “pink TOF” with VSD physiology who only received a VSD closure without need for augmentation of the RV outflow tract. However, we accounted for type of surgical repair in our models, thus it is unlikely that preoperative volume loading is driving this association.

In summary, we provide insight into RV remodeling during infancy and early childhood after TOF repair and report novel associations between a preoperative circulating biomarker signature and mid-term RV remodeling. Future studies will need to evaluate preoperative biomarker-RV remodeling associations, in addition to the role of preoperative extracellular matrix turnover on postoperative outcomes.

We acknowledge limitations in this study. This is a retrospective dataset with incomplete number of follow up echocardiograms by the fifth year after rTOF, which reduced the sample size. This could have introduced selection bias because patients that are followed elsewhere might be doing well clinically and not in need of follow up with echocardiograms at a quaternary institution. This attrition limits the robustness of medium-term outcomes and could affect the generalizability of the findings. Associations identified between preoperative biomarkers and strain measurements were modest, suggesting that these should be interpreted with caution. We present associations between biomarkers and postoperative function which do not imply causality. Mechanistic studies involving the use of tissue samples and advanced imaging modalities to characterize RV mass, volume, function, and fibrosis are necessary to enhance our understanding of the relationship between the preoperative biomarker signature and future RV remodeling. We acknowledge the difficulty in accounting for residual volume and/or pressure load to the right ventricle in a longitudinal study, as these are dynamic factors, however our sensitivity analyses indicate that residual pressure and/or volume loading did not counfound the biomarker-RVLS strain associations reported in this study.

## Conclusion

RV remodeling after TOF repair is a dynamic, multi-phased process, including a period of adaptation followed by a decline then stabilization in function by five years. These findings could be used to guide outpatient follow up, counseling, frequency of echocardiograms, and timing of the first cardiac MRI. Continued follow-up of this cohort will help determine when declines in RV function occur. A circulating biomarker signature indicating preoperative collagen turnover processes could help augment the knowledge of RV remodeling after TOF repair, which is complex given the change in loading conditions of the RV from the pre- to the postoperative period.

## Supplementary Information

Below is the link to the electronic supplementary material.

Supplementary Material 1

## Data Availability

No datasets were generated or analysed during the current study.
