# Supplementary material for: Association of preoperative Circulating biomarkers with echocardiographic measures of right ventricular strain five years after tetralogy of fallot repair
Source: Int J Cardiovasc Imaging. 2025 Oct 27;41(11):2229–39. doi: 10.1007/s10554-025-03533-4 (PMC12628454; doi:10.1007/s10554-025-03533-4)
Supplement: Supplementary file 1 — Supplementary Material 1 [file 10554_2025_3533_MOESM1_ESM.docx]

**Online supplement: Sensitivity analyses accounting for right ventricular dilation and pulmonary valve stenosis**

| **Association** | **Main model adjusted for PV stenosis** | **Main model adjusted for RV dilation** |
| --- | --- | --- |
| **RVLS**  MMP1  MMP9  ST-2  Gal 3  Nt-Pro BNP  PIIINP  PICP | **Unadjusted Estimate (95% CI), P value**  **-0.0276 (-0.0457, -0.0096)**, **0.0366**  -0.0072 (-0.0229, 0.0085) 0.3689  0.0165 (-0.0658, 0.0989) 0.6937  0.0527 (-0.2378, 0.3432) 0.7223  0.1938 (-0.0923, 0.4799) 0.1842  **-0.1781 (-0.3255,-0.0307) 0.0179**  **-1.1486 (-2.2094, -0.0878) 0.0338** | **Adjusted Estimate (95% CI), P value**  **-0.0244 (-0.044, -0.0047) 0.0149**  -0.0071 (-0.0222, 0.008) 0.3546  0.0075 (-0.0707, 0.0857) 0.8508  0.0463 (-0.2227, 0.3154) 0.7357  0.2072 (-0.068, 0.4824) 0.1401  **-0.1644 (-0.3072, -0.0217) 0.024**  **-1.1463 (-2.2099, -0.0827) 0.0347** |
| **FWS**  MMP1  MMP9  ST-2  Gal 3  Nt-Pro BNP  PIIINP  PICP | **-0.0183 (-0.0336,-0.0031) 0.0182**  -0.0034 (-0.019, 0.0128) 0.6846  0.0488 (-0.0395, 0.1372) 0.2786  0.1012 (-0.2268, 0.4292) 0.5452  0.1469 (-0.1562, 0.45) 0.3421  -0.1389 (-0.2911, 0.0134) 0.0738  -0.7967 (-1.9197, 0.3263) 0.1644 | -0.015 (-0.0308, 0.0008) 0.0628  -0.0031 (-0.0183, 0.0121) 0.689  0.045 (-0.0406, 0.1307) 0.3025  0.0853 (-0.2048, 0.3754) 0.5646  0.1689 (-0.1302, 0.468) 0.2685  -0.118 (-0.2641, 0.0281) 0.1134  -0.7645 (-1.8877, 0.3588) 0.1822 |
| **SR**  MMP1  MMP9  ST-2  Gal 3  Nt-Pro BNP  PIIINP  PICP | -0.0012 (-0.003, 0.0006) 0.1813  -0.0002 (-0.0012, 0.0008) 0.6756  -0.0001 (-0.0053, 0.0051) 0.9666  -0.0028 (-0.0215,0.016) 0.7711  0.0085 (-0.0115, 0.0285) 0.4049  -0.005 (-0.0169, 0.0069) 0.4116  -0.0485 (-0.141, 0.0441) 0.3045 | -0.0009 (-0.0029, 0.001) 0.3414  -0.0002 (-0.0011, 0.0008) 0.7146  -0.0006 (-0.0061, 0.0049) 0.83  -0.0043 (-0.0199, 0.0113) 0.5886  0.0125 (-0.0069, 0.0319) 0.2053  -0.0031 (-0.0141, 0.0079) 0.5819  -0.0422 (-0.1327, 0.0482) 0.36 |
| **FWSR**  MMP1  MMP9  ST-2  Gal 3  Nt-Pro BNP  PIIINP  PICP | -0.0004 -0.0023 0.0015 0.6711  -0.0001 -0.0012 0.0011 0.9152  0.002 -0.0019 0.006 0.3082  -0.0026 -0.0191 0.014 0.7603  0.0173 -0.0066 0.0412 0.1553  -0.0028 -0.014 0.0084 0.6236  -0.0402 -0.1552 0.0748 0.4936 | -0.0001 -0.0021 0.0019 0.9225  0 -0.0011 0.0011 0.9885  0.0016 -0.0023 0.0056 0.4172  -0.0041 -0.0173 0.009 0.537  0.0224 -0.0011 0.0459 0.0612  -0.0006 -0.0111 0.0098 0.9071  -0.0346 -0.1476 0.0784 0.5482 |

Sensitivity analyses of the associations between preoperative biomarkers and postoperative outcomes. Multivariable generalized estimated equations (GEE) models were adjusted separately for right ventricular (RV) dilation and residual pulmonary valve (PV) stenosis. RV longitudinal peak systolic strain (RVLS), free-wall peak systolic strain (FWS), peak systolic strain-rate (SR) and free-wall systolic strain-rate (FWSR), galectin-3 (Gal 3), Procollagen-type-I carboxy-terminal-propeptide (PICP), Procollagen-type-III-amino-terminal-propeptide (PIIINP), matrix-metalloproteinase-1 (MMP1), matrix-metalloproteinase-9 (MMP9), soluble suppression of tumorigenicity-2 (sST2), and N-terminal-pro-B-type-natriuretic-peptide (NT pro-BNP)
